# The Role of Emotions in Classroom Conflict Management. Case Studies Geared Towards Improving Teacher Training

**DOI:** 10.3389/fpsyg.2022.818431

**Published:** 2022-03-16

**Authors:** Ibis M. Alvarez, Montserrat González-Parera, Borja Manero

**Affiliations:** ^1^Department of Basic, Developmental and Educational Psychology, Universitat Autònoma de Barcelona, Barcelona, Spain; ^2^Software Engineering and Artificial Intelligence Department, Complutense University of Madrid, Madrid, Spain

**Keywords:** classroom climate, emotion, secondary school, conflict resolution, teacher training, virtual reality

## Abstract

The purpose of this paper is twofold: firstly, to explore the emotional aspects underlying classroom conflict management, and secondly, to apply these notions to the contrasted analysis of two case studies. Our findings underscore the importance of examining teachers’ emotional regulation to better understand their performance when dealing with conflicts that affect classroom climate. In the final section, we make suggestions for introducing this perspective into initial teacher training through the use of Virtual Reality, a scenario that would allow pre-service teachers to experiment, record and reflect on affective and attitudinal issues that are decisive for effective classroom conflict management.

## Introduction

Today’s society is characterized by a multiplicity of changes. The educational context is no stranger to this situation; as a reflection of society, changes in classroom composition and newly emerging forms of interaction can affect the climate of coexistence, since there is an issue that permeates educational settings as a social and inevitable condition in human relations: school conflict.

Despite the undoubted importance of teachers commanding the competence to manage classroom climate, according to recent reports pre-service teachers in Spain feel dissatisfied with the theoretical nature of training in this area and also consider the practical training they have received to be deficient (López et al., 2017; Özen and Yildirim, 2020). According to the TALIS report (OECD, 2020), less than half (40%) of Spanish teachers reported feeling prepared to manage a class. This study also reveals that Spanish teachers spend the longest time trying to maintain order in class. Likewise, a recent study on satisfaction with the training received by students of the Official Master’s degree in Teaching in Secondary Schools, at four major Spanish universities, revealed that the competence that pre-service teachers perceive as lacking the most is precisely that related to knowledge of classroom interaction and communication strategies and skills to promote coexistence, address disruptive behaviors and manage conflict in the classroom. At the end of the master’s degree, only 34% of respondents provided the highest rating, although this is an improvement compared to the 19.8% that had done so at the beginning (Sarcedo-Gorgoso et al., 2020).

Conflict management competence is frequently assessed through self-report questionnaires that inquire about preferred coping patterns without taking into account the communicative situations in which conflict takes place or the emotional and attitudinal variables underlying the decisions made by teachers to deal with it (Galtung, 2000; Jones, 2000). Thus, in terms of both the training and assessment of this competence, it seems necessary to design realistic learning scenarios in which teachers can experiment and reflect on their ability to manage the classroom climate in authentic communicative situations.

### Research Objectives

The purpose of this study is twofold: firstly, based on the literature, to identify the affective and attitudinal factors linked to the origins and management of classroom conflicts; secondly, from a qualitative-phenomenological approach, through case analysis, to integrate theory and practice to illustrate the contribution of this approach to a better understanding of the factors involved in the dynamics of classroom conflict in secondary school.

We believe that this analysis will provide conceptual and methodological bases for the design of teacher training actions in a scenario that promotes, on the one hand, awareness of behaviors in the face of conflict events and, on the other hand, the possibility of practicing more effective alternatives for classroom climate management. Based on the results that will be presented, at the end of the article ideas will be suggested to elaborate an experiential and experimental training proposal, through Immersive Virtual Reality (IVR).

## Theoretical Background

In this section we will begin by clarifying the definition of conflict in the educational setting. We will focus on various emotional and attitudinal factors that determine classroom conflict and its management. We will then proceed with a review of the literature that will help identify possible patterns of behavior that are usually displayed when dealing with conflicts.

### Conflict as a Communicative Experience

A widely held conception describes conflict as “an expressed struggle between at least two interdependent parties who perceive incompatible goals, scarce resources, and interference from the other party in achieving their goals” (Hocker and Wilmot, 2014, p.13). Giving greater weight to affective-motivational variables, conflict has also been described as a subjective experience: “To recognize that we are in conflict is to acknowledge that we have been triggered emotionally” (Jones, 2000, p. 91).

Aligned with the latter perspective, in this article we understand conflict as a particular type of communication, defined by Luhmann (1982) as an expressed contradiction that blocks communicative processes. By blocking communication, classroom conflicts infringe upon interpersonal relationships and can give rise to disruptive events (misbehaviors or critical incidents) that hinder the development of teaching (e.g., Bingham et al., 2009; Tripp, 2012). In the present study, these events are considered conflict situations, initiated by students that can dramatically affect the classroom climate.

We share the ecological approach to classroom climate proposed by Doyle (2006), understood as a communicative context with characteristic purposes, dimensions, features and processes, whose particularity has consequences for the behavior of occupants of that setting. From an ecological perspective, “management is a complex enterprise because order is jointly accomplished by teachers and students and because a large number of immediate circumstances affect the nature of orderliness, the need for intervention, and the consequences of particular teacher and student actions” (Doyle, 2006, p.100).

From this standpoint, there are several important dimensions of classrooms climate that are already in place when teachers and students arrive at the classroom door. These include Multidimensionality—a large quantity of events and tasks in classrooms takes place; many people, with different preferences and abilities must use a restricted supply of resources to accomplish a broad range of social and personal objectives; Simultaneity—many things happen at once in classrooms. While helping an individual student during seatwork, for instance, a teacher must monitor the rest of the class, acknowledge other requests for assistance, handle interruptions, and keep track of time; Immediacy—there is a rapid pace of classroom events; Unpredictability—classroom events often take unexpected turns, events are jointly produced and thus it is often difficult to anticipate how an activity will go on a particular day with a particular group of students; Publicness—classrooms are public places and events, especially those involving the teacher, are often witnessed by a large portion of the students; History—classes meet for 5 days a week for several months and thus accumulate a common set of experiences, routines, and norms, which provide a foundation for conducting activities for the rest of the term or year. All these factors combine to create demands and pressures on participants as activities are played out in these environments. These demands and pressures are placed especially on teachers who carry professional adult responsibility for planning and monitoring classroom activities. Ecologically, these pressures and demands are the origins of the task of classroom management. In most instances, therefore, teachers have little leisure time to reflect before acting (Doyle, 2006).

In addition, management demands are systematically related to the types of activities used in the classroom (Garrett, 2008; Wilkinson et al., 2020). Student work involvement or engagement is higher in teacher-led, externally paced activities than in self-paced activities. Involvement is also especially low during activities in which there are prolonged presentations. Thus, according to Doyle (2006), “the key to a teacher’s success in management appears to be his or her (a) understanding of the likely configuration of events in a classroom, and (b) skill in monitoring and guiding activities in light of this information” (p.116). Therefore, the effectiveness of classroom conflict management cannot be defined solely by stereotypical behavior patterns as traditional teacher education often suggests. Successful classroom management also involves aspects of the affective-attitudinal dimension that allow recognizing when and how to act to face conflict events in immediate circumstances (Evertson and Poole, 2008).

Attitude is an important concept to understand human behavior. More relevant to the present concerns is what this controversy regarding the attitude-behavior relation implies for a definition of attitude. Allport (1935), in his influential chapter for the Handbook of Social Psychology, defines attitude as “a mental and neural state of readiness, organized through experience, exerting a directive or dynamic influence upon the individual’s response to all objects and situations with which it is related” (p. 810). Beginning with these early tracings of the attitude concept, a large number of definitions have been offered.

Like many psychological variables, attitude is considered a hypothetical or latent variable, rather than an immediately observable variable (Fazio, 2007). From this view, Green (1954) argued that “the concept of attitude does not refer to any one specific act or response of an individual, but is an abstraction from a large number of related acts or responses” (p. 335). Zanna and Rempel’s (1988) formulation added that “the attitude may be based on appraisals of the attributes that characterize the object, as in expectancy-value frameworks” (p. 608). According to this view, any such automatic activation of the attitude is viewed as playing a critical role in the process by which an attitude may exert influence on information processing, judgment, and behavior. Indeed, from its outset, this theoretical conceptualization of attitudes has been embodied within a model attempting to specify the process(es) by which attitudes “guide” behavior (Fazio, 2007). This definition may help understand how attitudes can explain the strategies that teachers use to regulate the emotional experiences that are activated during the management of classroom conflict. These attitudinal aspects seem linked to actions that teachers take to create an environment that supports and facilitates both academic and social-emotional Learning (Emmer and Stough, 2001).

Emotion regulation refers to “the processes by which individuals influence which emotions they have, when they have them, and how they experience and express these emotions” (Gross, 1998, p. 275). The regulation of emotion is defined predominantly in terms of the conscious or volitional self-regulation of emotion. In other words, emotion regulation means the capability to manage the emotional experiences and expressions (Gross, 2002). As stated Evertson and Poole (2008), in relational interactions trust is a key component of an effective classroom. “Despite the best laid plans, student misbehavior will occur. Reactions to this misbehavior require careful planning to ensure a teacher’s responses are productive” (p.136).

## The Role of Emotions in the Origin and Management of Classroom Conflict

As Ekman (1985) and Damásio (1999) have convincingly demonstrated, the behavioral element of emotion is projected in the forms of expression of subjective emotional experience (facial expressions, tone of voice, gestures and body postures) that we communicate intentionally or unintentionally. Considering this primary condition, when conflicting events occur during interactions with students, some physiological changes and verbal and non-verbal emotional expressions will occur that will be consciously perceived by teachers and observed by their students. These signals trigger in both certain modes of action (Sutton, 2004; Keller and Becker, 2020).

Following Lazarus (1991), the emotional activation—tendency toward action—that we experience when facing conflicts could be caused by an incongruence with expectations or by the occurrence of an unforeseen event. This activation, in turn, instigates a personal interpretation (*appraisals*) of the communicative situation, which determines the qualities of the emotions that are projected, and these will affect the strategy implemented to manage the conflict. For example, a student’s defiant behavior can be appraised, with different resultant teacher emotional responses, as a threat to a teacher’s authority or as a sign of a student being over-challenged by the work task. Variations in appraisals occur because situations can be interpreted in different ways as a result of personal characteristics, social history and cultural expectations (Schutz et al., 2007).

Academic emotions refer to a set of emotions that are experienced by students and teacher in learning or teaching situations (Pekrun, 2006). They are short-lived and intense active states that arise in response to a particular stimulus. Academic emotional valence refers to whether the stimulus is pleasant or unpleasant, while academic emotional arousal describes the academic emotional intensity that a stimulus can cause. Based on this classification, emotions can be divided into four groups: positive arousal emotions (e.g., enjoyment, pride), positive emotions (e.g., enthusiasm, interest), passive arousal emotions (e.g., anger, anxiety), and negative emotions (e.g., frustration, depression) (Pekrun, 2006).

Emotional experiences were found to emerge during teacher judgments regarding perceived success (Schutz et al., 2007). Thus, teachers may experience happiness when an instructional objective is met or students follow directions, and pride comes when students excel or eclipse their peers or give a response or fulfill some tasks that teachers did not expect them to complete. They report frustration when students cannot grasp a concept; anger with misbehavior, disappointment with lack of effort, and anxiety when competence is challenged (Sutton et al., 2009; Cubukcu, 2013). These emotions often arise from management and disciplinary classroom interactions, and teachers report that they try to regulate these emotions frequently because they believe it helps them achieve their goals (Sutton, 2004).

Guilt is an unpleasant feeling due to the nature of caring and feeling responsible for students, and it is commonly felt by teachers who perceive they could not do what they were supposed to do and led to disappointment in others (van Veen and Lasky, 2005). Frustration and anger arise from several sources related to thwarted goals, including students’ misbehavior and violation of rules (Cubukcu, 2013). Teachers also become angry when they believe that students’ poor academic work is due to controllable factors, such as laziness or inattention.

Many teachers report that their anger and frustration lead to changes in their classroom behaviors and coping strategies. Intrusive thoughts make it difficult for them to concentrate on what they are doing before the emotion episode, and that students are the immediate target of the anger and frustration (Sutton, 2007).

Positive emotions such as joy, enthusiasm, gratitude, admiration, interest, satisfaction, optimism, and others lead to proactive attitudes that foster conciliation and collaboration (Montes et al., 2014). It has also been observed that, in critical situations, the activation of positive emotional states allows consideration and elaboration of plans for future action (Lyubomirsky et al., 2005), while the use of predominantly reactive strategies has been associated with teachers’ stress and emotional exhaustion (Clunies-Ross et al., 2008; Tsouloupas et al., 2010; Pedditzi et al., 2021).

Negative emotions, such as fear, anger, sadness, and guilt, are associated with reactive/adaptive behaviors that are activated in response to immediate events (Frijda, 1986; Sutton, 2007). Conflicts that emerge in teacher-student interactions are a source of these types of emotions. For example, Oplatka and Iglan (2020) found that primary and secondary school teachers reported experiencing some form of fear in interactions with their students during lessons. Two coping strategies emerged in the interviewees’ accounts, ranging from passive strategies that avoid directly confronting the source of the fear (e.g., emotional disengagement) to more active strategies that target the source of the fear (e.g., peer involvement, humor, etc.).

Positive and negative emotions are important activators of the attitudes that guide behavior, since a positive mood can facilitate creative, holistic and more flexible problem solving, whereas negative emotions can promote a more rigid and analytical way of thinking (Rockladge and Fazio, 2018). Deactivation emotions, such as boredom, disappointment, sadness or despair are detrimental to any deep treatment of information related to the teaching task, whilst relaxation and relief can reduce the attention (Pekrun and Schutz, 2007). As Emmer and Stough (2001) emphasize, from an ecological perspective on teaching, classroom management can be defined as any of the actions that teachers perform to maintain student attention, and through this, to create an environment that fosters both academic and social-emotional development.

Several studies (von Gilsa and Zapf, 2013; Taxer and Gross, 2018; Chang, 2020) showed that surface acting (e.g., hiding anger and fear) is significantly linked to emotional exhaustion, depersonalization, and inefficacy. Conversely, the teacher’s emotional authenticity fosters adaptive emotional reactions in students that is higher levels of enjoyment and lower levels of anger and anxiety (Keller and Becker, 2020).

In relation to anger, Chang and Taxer (2020) found that this attitude leads teachers to employ punitive strategies, especially when students’ misbehavior is seen as intentional and controllable. Although they may be effective in the short term, teachers’ coercive behaviors have been linked to the existence of escalating disruption-coercion-disruption loops (Martínez et al., 2020).

In contrast, as corroborated by Chang and Taxer (2020) in their study on emotion regulation strategies in response to classroom misbehavior, teachers who typically reappraise have the least negative affective experiences in the context of student misbehavior and are less likely to suppress their in-the-moment negative emotions. The prevalent premise of ‘Don’t show them!’ (Sutton, 2004, p. 379) when it comes to teachers regulating their negative emotions is to be welcomed because of the emotional contagion processes (Keller and Becker, 2020, p.13). These results are also consistent with several studies that have found that engaging in emotional perspective-taking allows teachers to react appropriately to disruptive behavior (Evertson and Weinstein, 2006; Barr, 2011). Similarly, teachers who expressed close relationships with disruptive students also described emotional perspective-taking, empathy, and emotion regulation (McGrath and Van Bergen, 2019). These characteristics are likely to be particularly helpful when forming relationships with disruptive students by guiding effective classroom management (Garner, 2010) and supporting a positive classroom climate (Jennings and Greenberg, 2009).

### Conflict Management Strategies From the Emotional Perspective

In order to assess the strategies that teachers use to manage classroom conflict, one of the most widespread models in educational research is the “Rahim” Model of Conflict Management (Rahim, 1983). This model describes conflict and negotiation processes by referring to two basic dimensions: concern for self (i.e., the degree to which they aim to address their own concern in conflict management processes) and concern for others (i.e., the degree to which individuals try to address the concern of the other party involved in a conflict).

On the basis of different combinations of these two dimensions, five strategies for managing interpersonal conflict in the teacher-student relationship have been distinguished: (1) *Integration* (e.g., reasoning with the student inside or outside the classroom; involving the student in individual and group settings to discuss the behavior that causes the potential conflict event); (2) *Compromise* (e.g., reasoning and discussing issues and problems with the student and/or with the whole class to explore new possible solutions and ways of dealing with the individual and relational difficulties that arose); (3) *Obliging* (for example, deliberately ignoring interruptions or minor infractions); (4) *Avoidance* (e.g., delaying discussion and confrontation about individual and relational difficulties that arose; sending student to see the principal); and, finally, imposing and authoritarian strategies, such as (5) *Domination* (e.g., issuing a verbal reprimand; asking the student to leave class; imposing sanctions) (Morris-Rothschild and Brassard, 2006; Montes et al., 2014; Doǧan, 2016; Claessens et al., 2017).

However, according to Galtung (2000), identifying behavior is not enough for effective conflict management. Conflict transformation requires attention to three interrelated elements, which the author called the “triadic model.” In this model, Galtung places the following three elements in a pyramid shape: (A) underlying *attitudes* of all the parties involved in the conflict toward the conflict issue; (B) overt *behavior* in relation to the conflict and interaction with the opponent; and (C) the *conflict* itself, which Galtung calls ‘contradiction.’

In short, identifying the role of emotions seems essential to better understand the nature of conflict. Emotional processing in all its complexity—including the components of emotional expression through verbal behaviors, the transparency of emotional states that are projected in non-verbal language (facial expressions, tone of voice and body posture) and the appraisal of emotional experiences driven by emotional arousal—provide clues to the choices teachers make when managing classroom conflict. The key is to recognize the inevitability of emotions in the face of conflict and to view them as a tool for managing conflict, rather than as an obstacle or something to avoid. The question is how this approach can be used to help teachers to better manage classroom conflict.

## Method

In order to meet the empirical objective of this research, we considered it appropriate to adopt a qualitative-phenomenological approach based on the study of contrasting cases. According to Miles and Hueberman (1994) by looking at a range of similar and contrasting cases, we can understand a single-case finding, grounding it by specifying how and where and, if possible, why it behaves as it does.

### Context of the Study and Case Selection

We selected two cases elaborated by pre-service teachers, based on the systematic and reflective recording of reported classroom interactions in secondary schools. Case elaboration and analysis is a practical task required in the first internship period, linked to the module Psychopedagogical and Social Training of the Master’s degree in Teaching in Secondary School at the Universitat Autònoma de Barcelona [UAB], (2021).

**FIGURE 1:**
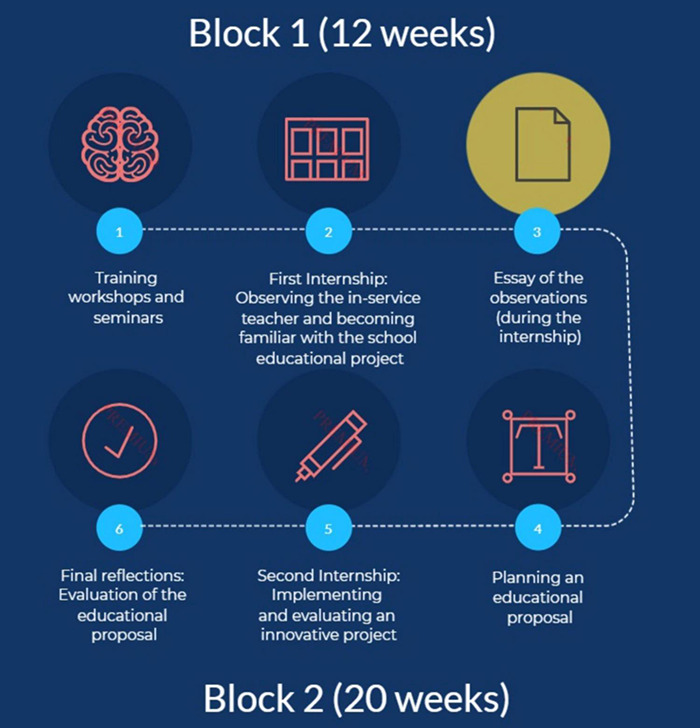
Practicum process in the Master’s degree in Teaching in Secondary School at the UAB.

Students do their internships in educational establishments under the supervision of a school mentor and a university practicum tutor, who will guide them in this first contact with the professional world in the field of education. Figure 1 shows the design of the process followed at UAB, including the learning activity on which this study is based.

During their first internships pre-service teachers should become familiar with the classroom dynamics and curriculum requirements of a particular class. They should pay special attention to the classroom dynamics of one of the observed groups, to the role of the teacher and to the teaching resources available. Previously, before the first internship period, students (pre-service teachers) are expected to participate actively in the guided activities (workshops, practicum seminars) and supervised activities (group and individual tutorials) offered in this master’s degree with the objective to help them prepare for their internship.

Specifically, the work assigned to pre-service teachers in the first stage (Block 1) consisted of carrying out a participant observation in a classroom (step 1, shown in Figure 1) during the first internship period (step 2). The observations were carried out simultaneously by two or three students who were at the same educational establishment. Educational establishments must be part of the school network promoted by the Department of Education of the Catalan Government. Priority will be given to schools and institutions with a skills-based curriculum aligned with the kind of teaching proposals promoted in this master’s degree. The pre-service teachers were asked to produce a reflective description of the development of the lesson they observed in the form of a narrative essay (step 3).

The essay is structured into four sections. The first section consists of a description of the context in which the observed activity takes place, making reference to the characteristics that may influence everyday life, teaching and learning and a brief presentation of the pupils in the classroom, focusing on their learning processes, and of the teacher, focusing on his/her teaching processes, as well as a description of the educational spaces (e.g., classroom organization). The second section describes the *organization of the observed activity* (topic, general competences and objectives, methodology, resources, organization of the space, etc.). The third section details the observations during the *development of the activity*. Finally, the preservice teacher is required to write a *reflection* on the observed students’ prospects of future development in the adolescent stage. Given the interpretative nature of this section, its content has not been included in the analysis presented in this paper.

Supplementary Appendix 1 shows the observation guidelines, in the form of guiding questions, which are given to pre-service teachers to help them record the required evidence during the first internship period. They must actively engage in the tasks set by their school mentors. They also need to collect evidence of the work done at their host school/educational establishment both to include in their portfolios and to have sufficient data for the educational proposal that pre-service teachers will need to design and implement in their next internship period (steps 4, 5, and 6, shown in Figure 1). Pre-service teachers’ performance at the host school and the quality of their essay will be also taken into account to assess this first block. The narrative essay with the recorded observations is assessed by the student’s academic tutor at the university. This assessment takes into account the quality of the evidence presented, as well as the student’s argumentation of his/her interpretation. School mentors will assess the preservice teachers’ behavior in this first internship period. To pass the course students are also expected to display a professional attitude and professional skills such as respect, cooperation, punctuality and active listening and participation. In any case, students are expected to respect the deontological ethics of the teaching profession.

The first and second authors of this paper have been teaching this module for several years. For this study, we selected two essays that narrate classes observed in state schools located in the metropolitan area of Barcelona, which has high percentages of immigrant and working-class population. The class groups were made up of boys and girls of this social profile, between 15 and 16 years of age, in the 4th year of the 2nd education cycle—the last level of Compulsory Secondary Education in Spain. In both cases, the lessons developed a contemporary history topic and were taught by teachers (all female) who were experienced in the subject matter. The difference between the two cases was the methodology used to teach the content and to manage the disruptive events that occurred during the class (please, see Supplementary Material with translation of the observation records: Case A and Case B).

The schools, students and teachers who supported the pre-service teachers during their placements where these observations were made were informed of the prospect of having evidence of their work published for academic purposes and anonymously, thereby gaining their explicit consent.

### Procedures and Data Analysis Processes

In this study we adopted the ecological approach to classroom climate, through which classroom activity during a lesson is conceived as a behavior setting composed of interactive segments. In simple terms, following Doyle (2006), the easiest way to understand the concept of segments is to think of them as a set of classroom “chronicles” or narrative records. “A classroom chronicle is a reasonably complete description of the behavior stream […] that contains information about scene coordinates (i.e., the participants, physical arrangements, props, and time) and a running account of action sequences within scenes (p. 100).”

**FIGURE 2:**
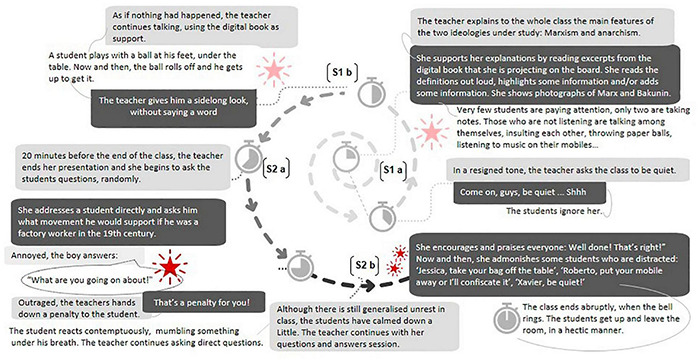
Causal network: Case A ‘The tale of the digital book.’

**FIGURE 3:**
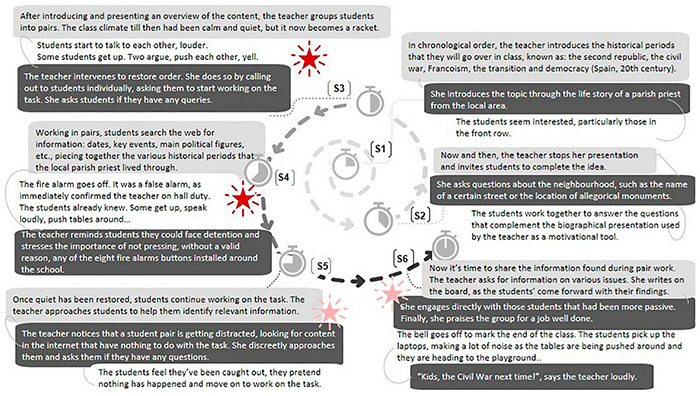
Causal network: Case B “The story of the local parish priest.”

Accordingly, we began our analysis by delimiting the segments of the classes reported in the two case studies. The segmentation was established based on changes in the following dimensions: (a) patterns for arranging participants, (b) resources used or sources of information, (c) roles and responsibilities for carrying out immediate actions, and (d) rules of appropriateness (i.e., the types of behaviors that are allowed and disapproved). According to Doyle (2006), a change in one or more of these dimensions represents a potential change in the nature of the situation in which students and teacher work. Two segments were delineated in Case A and six in Case B (see Figures 2, 3).

### Summative Content Analysis

In the first stage, we conducted a summative analysis of both cases. Summative analysis offers the opportunity to embrace the research subject while involving teams of co-researchers. Whereas the principal researcher has overall accountability for the study, the co-researcher takes on a commitment to be fully involved in all analysis sessions. By doing so, co-researchers must be aware of the importance of the collaborative aspect of the method and of developing a negotiated understanding of a text (Rapport, 2010). Thus, to ensure internal agreement, decisions regarding collapsing and combining categories were conferred with the second author (an associate professor in educational psychology). Coding discrepancies were discussed and agreements about coding and modifications to the coding scheme were reached to mediate ambiguity in the coding scheme going forward.

In a second stage, seeking in-depth understanding and explanation, we identified in each of the delimited segments the dimensions of the triadic model proposed by Galtung (2000): (A) underlying affects and attitudes toward the conflict issue; (B) teacher behavior toward the conflict and his/her interaction with the student(s) giving rise to the conflict situation; and (C) the conflict itself, which consists of the “contradiction” perceived by the teacher. At this stage, following the cross-case analysis procedure outlined by Miles and Hueberman (1994), we used an analysis strategy geared toward transcendent themes and categories. Specifically, we implemented a summative content analysis (Rapport, 2010), which is based on definitions derived from the literature review. Table 1 summarizes the delimited categories and their corresponding indicators. The cases are then presented, followed by the corresponding analysis.

**TABLE 1:** Topics, categories, and indicators that guided the summative case analysis.

Topics	Categories		Indicators
Emotional experience during the conflict	Teacher emotions		Positive emotions (e.g., happiness, enthusiasm, interest……) Positive arousal emotions (e.g., enjoyment, pride) Negative emotions (e.g., disappointment, frustration, guilt) Negative arousal emotions (e.g., anger, anxiety) Deactivation emotions (e.g., boredom, sadness, despair)
	Attitudes		Open, flexible, empathetic (i.e., leaner-centered classrooms). Closed, rigid attitudes centered on self-interest (i.e., academically focused classrooms)
Behavior in the face of conflict *(Coping strategy)*	Active orientation	Domination	Use of position of power, verbal domination, perseverance; making confrontational statements (e.g., overt rejection; involves the administration, aggressive questions, humiliates, …)
		Collaboration	Open communication to explore the disagreement, identify underlying concerns and look for alternatives to satisfy each party’s interests (e.g., clarifies the situations by listening to a student, asking additional questions…).
		Compromise	Reasoning and discussing issues and problems with the student and/or the whole class in order to explore new possible solutions and ways to deal with the perceived relational difficulties (e.g., tell a student that you will talk to him after class …).
	Passive Orientation	Avoidance	Denying the existence of conflict, avoiding it or avoiding certain issues; making non-committal and/or irrelevant statements, etc. (e.g., make a joke, take no comment, do not speak on the subject …).
		Obliging	Acting kindly or altruistically, meeting the other person’s demands despite preferring not to do it (e.g., apologies, make a compromise, propose compensation…)

*Source: own construction based on the literature review (Rahim, 1983; Ellis and McClintock, 1993; Lyubomirsky et al., 2005; Morris-Rothschild and Brassard, 2006; Pekrun, 2006; Clunies-Ross et al., 2008; Evertson and Poole, 2008; Sutton et al., 2009; Costas et al., 2010; Cubukcu, 2013; Montes et al., 2014; Doǧan, 2016; Ciuladiene and Kairiene, 2017; Claessens et al., 2017; Chang and Taxer, 2020).*

## Findings

Following the analytical strategy described above, in this section we will now present the most salient findings from the analysis of the case studies. We will start from the summary produced in the first stage of the analysis and then present a more detailed analysis, derived from the categorization of the content in the second stage of the study.

### Undertaking Summative Analysis

The summative analysis began with a written summary produced by the researchers and which was written in response to the raw material provided by the narrative account (essay) of the two lessons observed by the pre-service teachers during their placements. The aim of this first stage of the analysis was to agree on a condensed, but rigorous, text that would allow the researchers to begin to consider what might be the essential content of each case, highlighting the affective motivational aspects present in the narrative accounts. The agreed texts are shown in Boxes 1, 2.

**Box 1. Key aspects of summative analysis of Case A “The tale of the digital book.”**

Two segments were delimited in this class, and the main difference between them was only the class organizational pattern. The first segment, which took up three thirds of class time, consisted of an expository monolog by the teacher, who supported her explanations by reading excerpts from the digital book, which she had projected on the classroom board. Multiple disruptive events occurred throughout the lesson, forcing the teacher to stop her talk to try to regain attention, unsuccessfully.

Fifteen minutes before the end of the class, the teacher promoted student participation in the construction of knowledge. She is the one who initiated the interactions, following the question-answer instructional scheme. The most critical incidents occurred during this second segment of the lesson.

Seen globally, the conflict events escalated as the lesson progressed, from more or less explicit student’s lack of interest during the first segment to open confrontation in the teacher’s interactions with the students. In dealing with conflicts, she implemented predominantly reactive and punitive strategies. She yelled angrily at students who “misbehaved” and deliberately embarrassed students who were disrupting the class. In terms of emotions, frustration and anger prevailed throughout the class.

**Box 2. Key aspects of summative analysis of Case B “The tale of the local parish priest.”**

In this lesson, six segments were delimited. During the first two segments, the teacher introduced the topic, managing to capture the students’ attention. She presented the lesson content linking it to the life story of a parish priest who worked in a parish very close to the school. Up to this point, no potential conflict events were identified.

From the third segment onwards, the teacher transferred the responsibility for learning to the students. Students worked in pairs while the teacher walked around the classroom to offer individual and timely help.

By focusing her attention on the needs of some student pairs, the teacher missed other potential conflict situations, for example: a fight between two students in segment three; the group being called to evacuate the class, in segment four, due to a false fire alarm, activated by some students in the class; two students’ lack of interest in the task, as they were caught using the Internet for unrelated purposes; and some students’ apathy when asked to contribute to the collaborative construction of knowledge. These events affected the classroom climate, causing disruptions and disturbances. When the teacher noticed this, she stopped the class and was proactive; she proposed preventive and conciliatory measures that appealed to collaboration; competent communication with all students was observed, reprimands/corrective statements were issued in a non-threatening manner.

This teacher maintained close relationships with her students from start to finish; she was energetic, enthusiastic and empathetic.

In this first analysis, we can clearly see two opposing cases in terms of how the lessons were conducted and conflicts managed. The teacher in “Case A” monopolized the class. She adopted the teacher-centered classroom paradigm, in which management is a form of oversight and students are allowed limited responsibilities. The classroom interaction followed the specific pattern of teacher initiates a question, student responds and teacher evaluates the response (Trigwell et al., 1999). In a reactive manner, the teacher occasionally stopped at the most critical incidents, calling for order from a position of power that left no room for negotiation. In contrast, the teacher in “Case B” made strategic use of different discursive resources, both to present the content and to manage disruptive behaviors during the lesson. She adopted the learner-centered paradigm (Reigeluth et al., 2017), facilitating collaborative and self-regulated learning, and providing authentic learning experiences, such as how to understand historical events through enquiry into the life of a person relevant to the community. She used varied instructional methods; transitions were taught and managed well. When she gave students control of the learning activity (cooperative work, in pairs), she exercised her authority in a democratic climate, being sensitive to the students’ needs and interests.

### Going Into Details: Summative Content Analysis

In order to gain a deeper understanding of the management of conflicts that affect classroom climate, in the second stage of our study a more detailed analysis of both cases was carried out, based on the triadic model proposed by Galtung (2000). In this analysis, we explored the role that the affective-attitudinal component may be playing in the decisions taken by both teachers to manage the conflict events that occurred during class (Please see Supplementarty Appendix 2 for an example of this first codification in an interactive segment in both cases). We were interested, on the one hand, in identifying the contents related to each of the categories that make up the model, and on the other hand, in identifying a possible causal relationship between them (causal network).

### Case A “The Tale of the Digital Book”

Figure 2 shows the causal network suggested by the detailed analysis of Case A. Specifically, a spiral format represents the interactions between the teacher and the students in the two segments (S) identified in the analysis of this lesson. As previously noted, following the ecological classroom approach, the segments were delimited based on changes in the four dimensions suggested by Doyle (2006): (a) patterns for arranging participants (e.g., expository format focusing on teacher presentation to the whole class vs. supervision of group work); (b) resources used or sources of information (e.g., books vs. computers); (c) roles and responsibilities for carrying out immediate actions (e.g., individual vs. collaborative work); and (d) ‘rules of appropriateness,’ i.e., the types of behaviors that are allowed and disapproved (e.g., speaking vs. keeping quiet). The letters *a* and *b* in S1 and S2 indicate slight variations in the format of interactions, caused by disruptions.

We will now present the agreed content regarding all aspects of the communicative situation in which conflicts arose in Case A, according to the dimensions of the triadic model proposed by Galtung (2000). We will focus specifically on the teacher’s emotional experience (attitudes and affects) and her behavior in terms of conflict management (contradictions).

#### Contradictions

In all the segments delimited, conflict situations of disinterest, disruption, distraction and rebellion arose due to contradictions between the teacher’s expectations with regard to monitoring the class and participation.

#### Attitudes and Affects

In most conflict situations, the teacher acted reactively, apparently driven by frustration, tension, anger and irritation caused by the contradictions experienced. Frustration arose, for example, when she observed that very few students were actually listening to her. When a student played with a ball (Segment 1b), she responded with a glare, a reactive gesture full of anger. She also showed frustration when another student retorted “What are you going on about!” (Segment 2a). At the end of the class, she acted with indifference in front of a class that was getting agitated by her continuous reprimands. At this point, the teacher had lost control of a class that ended up collapsing due to multiple and successive conflicts that prevented her from moving forward.

#### Behavior in the Face of Conflict

To deal with conflicts in this class, the teacher mostly adopted a domineering style, accompanied by a rigid attitude, centered on personal interest and protected by the teaching role exercised in an autocratic manner. Occasionally, she managed to regain control in a coercive manner, imposing sanctions on the student who responded evasively and contemptuously to her invitation to enter the historical scenario that she tried to transfer from the digital book to the figurative experience (segment 2a). From this position, the strategies used to deal with the inevitable class disruption led to blocking conflicts, which remained latent and eventually escalated, causing the class to collapse just as it was supposed to end. According to Galtung’s (2000) theoretical model, in this case the teacher opted for resolution actions. Instead of engaging in dialog to transform the communicative situation, she only managed critical conflicts, with specific, reactive and punitive actions. Poor class management led to an abrupt closure, with no time to point out the continuation of the didactic unit, in front of a class that was eagerly awaiting the moment to escape from the classroom.

### Case B “The Tale of the Local Parish Priest”

The results of the analysis of Case B are presented below. Figure 3 shows the causal network suggested by the in-depth analysis, focusing on the lesson development, which in this case ran through six segments (S).

As in the previous case, following a detailed analysis of the dynamics of the communicative situations throughout the lesson in Case B, we will now summarize the agreed content regarding conflict management, following the triadic model proposed by Galtung (2000).

#### Contradictions

In this class, potentially conflict situations, associated with distraction and disinterested behavior, arose from segment three onwards, when the class organization changed (students began to work in pairs).

#### Affect and Attitudes

Unlike Case A, in this case the teacher made time to deal with all the conflicts that occurred, even those that were latent in the last two segments. In all situations she acted proactively, displaying an attitude that was open to dialog and willingness to collaborate. In general, she was able to show empathy and regulate her emotions at all times, even when the most disruptive incident involving the whole class occurred: the false fire alarm (incident alluded to in the fourth segment). At this point, she explicitly showed her anger and openly condemned what happened, in a reactive manner, asserting her authority. Having negotiated with the students, she resumed her lesson and was able to reinstate the cooperation climate needed to achieve the educational aims.

#### Behavior in the Face of Conflict

In conflict situations, this teacher mainly adopted non-confrontational strategies, such as seeking compromise and being obliging/accommodating. For example, in segment three—when the false fire alarm went off after students’ complicity—and in segment six, when faced with students’ apathy when asked to contribute to the collaborative work requested of the whole group. In both situations, the teacher used persuasive strategies, discussed the issue with the students to explore new possible solutions and ways of dealing with individual and/or relational difficulties that could lead to further conflict. In the fifth segment, she offered personalized help to the students, explored with them alternatives to solve difficulties that might be limiting their effective participation. These strategies made it possible to successfully overcome conflicts, even in the most critical situation, when the false fire alarm went off. At the end of the lesson, the teacher left the way open for the next lesson with a motivational message: “Kids, the Civil War next time!”.

## Discussion

This paper set out to meet two main objectives. First, to apply the theoretical foundations linking emotion and conflict processes to the educational context. Additionally, by contrasting case studies, we wanted to illustrate the contribution of this approach to a better understanding of all the elements present in the dynamics of classroom conflict and its management.

### Three Essential Ideas for Understanding Classroom Conflict From an Emotional Perspective

We will now discuss the premises backed up by our study, based on the empirical findings presented in the previous section.

Firstly, conflict can be understood as a communicated contradiction that can block communicative processes (Luhmann, 1982), depending on the emotional experience it promotes (Jones, 2000). This idea is furthered by Galtung (2000), whose theory has shown that for conflict transformation it is not enough to focus on behavioral elements, such as coping patterns. Galtung insists on the need to identify the non-visible elements in a conflict situation, such as the attitudes of all parties toward the conflict issue, as well as to discover the origin of the contradictions that cause it. As pointed out in the introduction, usually only one or two parts of the triad are addressed in teacher training, such as the contradiction (the conflict itself) and the behaviors displayed by the opponents. What is largely ignored are the motives and attitudes underlying the reaction to the conflict. As suggested by Fazio (2007), in order to understand the role that attitudes play in determining behavior, it is important to look at the process, motivations and affective experiences involved. “In particular, negative attitudes promote avoidance behavior. In contrast, a positive attitude encourages approach behavior, which creates the possibility of information gain and a more nuanced understanding of the object” (Rockladge and Fazio, 2018, p. 517).

Secondly, and in relation to the above, regarding we would like to highlight the role of emotions in the origin and management of conflict. As Jones (2000) notes, we know that we have entered a conflict because we get the feeling that something is not going well. This perception is linked to the imminent involuntary emotional expressions that are activated when we face in tense situations such as an interpersonal conflict. In classroom interactions, these verbal and non-verbal expressions are not only consciously perceived by teachers, but they are also observed by their students (Keller and Becker, 2020) and they result in ways of action that are more or less geared toward resolution, depending on the nuances of emotional activation that takes place in the interactions surrounding conflict management (Lazarus, 1984; Chang and Taxer, 2020). Our results are in in line with previous reports that showed these emotional experiences often arise from management and disciplinary classroom interactions, and teachers report that they try to regulate these emotions frequently because they believe it helps them achieve their goals (Sutton, 2004; van Veen and Lasky, 2005; Schutz et al., 2007; Sutton et al., 2009; Cubukcu, 2013).

Thus, positive affective states lead to proactive responses (Montes et al., 2014), while negative affective states are linked to reactive responses ranging from dominance to avoidance (Oplatka and Iglan, 2020), as observed in the analysis of Case A. This notion fits in with the ecological approach to classroom climate. From this perspective, according to Doyle (2006), successful classroom management also involves aspects of the affective-attitudinal dimension, which is manifested in the way the teacher organizes and monitors the different events that make up the lesson. In this sense, the decisions teachers make determine when and how they act to deal with conflict events, with the immediacy that classroom climate management demands. In our study, the differences between the pedagogical approaches used by the two teachers help to understand the greater or lesser difficulty they had in managing the classroom. The amount of time teachers spend organizing and directing students, interacting with individual students, and dealing with inappropriate and disruptive behavior is linked to the type of activity and the physical arrangements of the setting (Doyle, 2006). Studies suggest that the greater the amount of student choice and mobility and the greater the complexity of the social scene, the greater the need for overt monitoring and managing actions by teachers (Garrett, 2008; Wilkinson et al., 2020). Positive teacher-student relationships are seen as the very core of effective classroom management, as confirmed in our analysis of Case B. In contrast, as found in Case A, the external reward and punishment strategies are not seen as optimal for promoting academic and social emotional growth and self-regulated behavior (Evertson and Weinstein, 2006; Evertson and Poole, 2008).

Finally, the third premise that our study ratifies complements the two previous ones and leads us to understand conflict management strategies from an emotional perspective. In this regard, we find the reinterpretation of the Dual Concern Model (Rahim, 1983) convincing. This approach states that people choose different ways, different strategies, to manage conflicts based on two primary motivations or interests: concern for self and concern for others. In classroom climate management, this notion is linked to the pedagogical approach that the teacher uses to develop the lesson, which may be centered around the teacher’s authority to impose what and how to learn (Trigwell et al., 1999) or on the students’ interest and abilities to construct knowledge, guided by the teacher (Reigeluth et al., 2017). Teachers regarded the use of an effective teaching method as a prerequisite to cope with inappropriate behaviors while managing their classes (Martínez et al., 2020; Özen and Yildirim, 2020). The first approach, a teacher-centered classroom, as evidenced in Case A “The tale of a digital book,” leads to reactive strategies geared toward resolution and characterized by domination or avoidance; the second approach, student-centered learning, as observed in Case B “The tale of the local parish priest,” results in proactive strategies based on dialog, such as the integration of viewpoints and compromise with new ways of acting to overcome conflict, in the short and long term.

### Classroom Conflict Dynamics Through the Prism of Galtung’s Triadic Model

Our second objective, of a practical nature, was to apply the theory to the analysis of two different cases of classroom conflict management in a lower secondary school. The results of this analysis allowed us to identify the role that the affective-attitudinal component may be playing in the decisions taken by the teachers in both cases to manage the conflict events that occurred in class.

Beginning with the interpretable aspects, such as the contradictions that cause conflicts, in our study we found that the conflicts were mostly non-violent, disruptive events that affected the classroom climate and occurred occasionally when teachers’ expectations regarding class monitoring and student participation failed (Evans et al., 2019).

In relation to the affective-attitudinal component, as Galtung (2000) argues, the appraisal of emotional experiences driven by emotional arousal allows us to understand the decisions the teachers made to manage classroom conflicts. Thus, in Case A, we found causal relationships between attitudes of anger and frustration and coping strategies based on domination (repeated verbal reprimands or the imposition of sanctions) or avoidance (delaying the discussion or intentionally ignoring the confrontation). This link is consistent with findings from similar studies mentioned in the literature review (Pérez-Fuentes, 2011; Chang and Taxer, 2020).

We also found that the use of predominantly reactive or surface acting strategies (e.g., hiding anger and fear), such as those observed in the teacher in Case A, can lead to teacher stress and emotional exhaustion (Clunies-Ross et al., 2008; Tsouloupas et al., 2010; von Gilsa and Zapf, 2013; Chang, 2020; Pedditzi et al., 2021).

On the contrary, emotional perspective-taking, empathy, and emotion regulation would have allowed the teacher in Case A to establish a constructive dialog with the students involved in the conflict communicative situations, leading to a more effective classroom climate management, as demonstrated by several studies cited previously (Jennings and Greenberg, 2009; Garner, 2010; McGrath and Van Bergen, 2019).

These notions were confirmed in the study of Case B. The teacher’s attitudes to conflict events denoted enthusiasm and empathy, which led to the use of collaborative strategies such as engagement (reasoning and discussing issues and problems with the student and/or the whole class to explore possible new solutions and ways of dealing with the individual and relational difficulties that arose) and integration (involving students in reasoning about the causes of the potential conflict event), even including being indulging, a compliant coping style with the other party, such as deliberately ignoring disruptions or minor infractions. Similar links were found by Montes et al. (2014) in a study based on self-referrals. Moreover, as also noted by Lyubomirsky et al. (2005), these strategies prevented the escalation of conflicts and enabled the teacher in Case B to timely transform the attitudes and behavior of students involved in the conflict events that emerged during class.

In summary, in line with arguments by Galtung (2000) and Jones and Bodtker (2001), in this study we have corroborated that a full appreciation of the elements presents in the conflict communicative situations that emerge during class facilitates their understanding. Understanding regulation in terms of conscious or volitive self-regulation (Gross, 1998, 2002), becoming aware of emotional experiences will help teachers to regulate their emotions during conflict management. In particular, identifying affective and attitudinal states and the nature of the strategies that are implemented to manage conflict could contribute to the re-evaluation necessary to change one’s own emotional experience—a process without which complex and effective conflict resolution is not possible—and this would consequently lead to better classroom climate management by teachers (Evertson and Poole, 2008).

## Conclusion

This study allowed us to draw at least the following conclusions.

Firstly, we believe that exploring the affective attitudinal aspects underlying classroom conflicts presents opportunities for teachers to learn how to manage them productively, but this is not enough. Successful classroom management also involves cognitive dimensions such as understanding and interpretation, skills that are necessary to recognize when and how to act to deal with conflict events in the classroom (Sutton, 2004; Chang and Taxer, 2020).

Secondly, and linked to the above, based on the ecological approach to classroom climate (Doyle, 2006), our study provides evidence that confirm the teachers’ actions in highly taxing situations involve making immediate decisions. In this regard, teachers require training to recognize the affective cues that trigger various reactive and ineffective automatic response patterns, and to become proactive in implementing strategies to reduce the impact of these triggers, thereby increasing their sense of efficacy (Evertson and Poole, 2008; Zee and Koomen, 2016).

Finally, given that emotions are considered inseparable from the educational context in which they emerge, paying attention to explanations of significant emotional experiences after they have occurred can help teachers identify and characterize emotionally relevant “courses of action” developed in the classroom for classroom climate management (Marsick and Sauquet, 2000; Evans et al., 2019).

The present study has some limitations and, accordingly, we suggest additional directions for future research. The data analyzed in this paper come from the reported observations of interactions in two secondary school classrooms performed by preservice teachers in their first contact with an educational institution. Although guided, consensual and supervised, the written narratives may be incomplete and may even contain interpretive biases. Future studies may expand the sample of cases, including a wider variety of disciplines and socio-educational contexts. In addition, it would be interesting to complement the observational records with interviews with students and teachers in order to investigate the experiences and motivations underlying the emotional experiences provoked by classroom conflicts. These options would minimize the high level of inference created by the analysis of reported narratives by 3rd parties, as well as make an ethical commitment to the participants, whose voices remain absent in this work.

Other data sources, such as descriptions of conflict cases reported by in-service teachers, could provide more authentic content. Expert teachers with academic standing (“good teachers”), teacher trainers and educational counselors could also provide information on the characteristics of the most common conflict situations in lower secondary schools, possible causes and ideas about more and less appropriate coping strategies.

### A Future (and Futuristic) Proposal for Teacher Training

Taking into account the practical and ethical limitations of simulating classroom conflict, we intend to explore, as an immediate line of research after this study, how immersive Virtual Reality (VR) training could improve teachers’ communicative competence through challenging and personalized virtual scenarios, while providing accurate information about which emotions are involved (positively or negatively) in classroom conflict management in a safe environment.

Before discussing the reasons why we consider the use of VR in this scenario, we should recall the concept of the magic circle, introduced by Johan Huizinga in his book Homo Ludens (Huizinga, 2002), which is a key concept in game studies. The magic circle is an imaginary place in which what happens inside the circle has no consequences outside the circle. Within the circle, the rules of the game apply. It is a safe place where one can fail without fear of affecting “real life.”

Virtual Reality is a perfect example of the magic circle, a place where you can “get it wrong” and repeat it as many times as you want. VR places the user inside a 3D artificial world in which he/she can interact with the environment as if it was real, allowing the creation of realistic scenarios (Ke et al., 2020). These realistic scenarios could place the teacher in an authentic classroom situation in which it would be essential to interact with a group of students (represented in avatars) and make multiple decisions immediately to achieve a classroom climate favorable to the achievement of educational goals (Doyle, 2006). This would be an ideal scenario to implement the much-needed practical training, in which teachers can experiment and reflect on their ability to manage the classroom climate.

The three distinguishing features of VR are “Interaction-Immersion-Imagination.” These characteristics are believed to facilitate experience specific and contextualized learning while increasing student motivation and commitment (Ke et al., 2020). Nonetheless, probably the biggest advantage of VR is that it allows users to embody learning experiences in such a way that it produces intense and real emotional sensations (Stavroulia et al., 2019). This feature helps teachers feel they are learning within a real environment, from an ecological learning perspective that enhances and fosters the transfer of knowledge, professional skills, and real-life management models. Only in a realistic environment can the users face the fear that newly qualified teachers experience when facing students (Oplatka and Iglan, 2020).

Based on the results obtained in this study, we find three very powerful reasons to favor VR for the training of future teachers. First, the communication blocks that occurs during a conflict can be realistically recreated in the virtual environment so that future teachers can learn to unblock them. Second, in order to recognize conflict, there must be a feeling that something is not right (Jones, 2000), which can only come about through direct experience. The possibility of recreating realistic environments can make it possible for the user to get such a feeling. Third, through effective feedback to the user, virtual reality allows the training of the most effective communication strategies depending on the type of conflict generated. For example, a situation could be simulated in which, after having driven the teacher to anger, he or she is taught to practice strategies other than domination or avoidance.

Regarding this last point, it is worth highlighting the capacity of current technologies for emotion detection, as studies in other fields, such as medicine, have shown (Kazemitabar et al., 2021). As noted in the introduction, the behavioral element of emotion is projected in facial expressions, tone of voice, gestures and body postures. If we add to these the content of the message or even the user’s biometric measurements (such as heart rate, skin conductance or pupil size), machine learning techniques can allow us to detect the emotions felt by the user in real time. For example, we could tell if the future teacher is angry and adapt the simulation to this situation accordingly.

The use of virtual simulations is increasingly seen as an opportunity to provide pre-service teachers with unique opportunities to experience examples of classroom life in a controlled and structured manner (McGarr, 2021). However, we believe that, in addition to VR, the Virtual Learning Environment (VLE) must incorporate technologies capable of detecting users’ emotions. In the VLE, rather than mechanically executing an actual sequence of instructional events, learning involves dynamic and complex interpersonal interaction skills. Thus, the experiences provided by VR learning can foster reflective and critical learning about effective classroom climate management.

Future research should also aim to examine the short- and long-term outcomes associated with emotionally generated conflict management approaches in a variety of conflict situations to be presented in context. Detailed observations of classroom interactions, such as those described in this study, will allow the creation of the algorithms responsible for modeling the behavior of the avatars that will form part of the virtual world. In this regard, it is important to carry out a broader study of recurrent critical incidents in secondary school classrooms, which can be provided by the pre-service teachers themselves and by expert teachers. Otherwise, a virtual classroom would be created where students would not present realistic behaviors, the virtual world would not be credible to the user and disengagement would occur. Moreover, VR simulated cases for performance analysis in a playful learning context do not compromise assessment and reputation. This can be very enriching for teachers, who often lament the theoretical nature of their training and ineffective practical approaches, where they have no opportunity for self-reflection or obtaining constructive feedback on their own performances.

## Data Availability Statement

The raw data supporting the conclusions of this article will be made available by the authors, without undue reservation.

## Author Contributions

All authors listed have made a substantial, direct, and intellectual contribution to the work, and approved it for publication.

## Conflict of Interest

The authors declare that the research was conducted in the absence of any commercial or financial relationships that could be construed as a potential conflict of interest.

## Publisher’s Note

All claims expressed in this article are solely those of the authors and do not necessarily represent those of their affiliated organizations, or those of the publisher, the editors and the reviewers. Any product that may be evaluated in this article, or claim that may be made by its manufacturer, is not guaranteed or endorsed by the publisher.
