# Supplementary material for: The Role of Emotions in Classroom Conflict Management. Case Studies Geared Towards Improving Teacher Training
Source: Front Psychol. 2022 Mar 16;13:818431. doi: 10.3389/fpsyg.2022.818431 (PMC8967289; doi:10.3389/fpsyg.2022.818431)
Supplement: Supplementary file 1 [file Data_Sheet_1.PDF]

Supplementary material with translation of the observation records: Case A and Case B

Case A

Class observation and analysis of class dynamics in a Year 4 group (Author: JEG, teacher IAV) (December 2018)

I. Description of the context in which the learning activity takes place

The secondary school XX [information omitted to maintain confidentiality] is located in the L'Hospitalet district, in one of the working-class neighbourhoods that were formed in this area of Barcelona after a wave of immigration in the 1960s.

The school currently has 32 teachers and two class groups per year group (from Year 1 to Year 4 of secondary education). In addition, there is a Special Education Support Unit which caters for eight students. The mixed-sex school's 350 pupils come mainly from a neighbourhood in the district of L'Hospitalet. The majority of these students are foreign-born.

The activity under observation was carried out in a Year 4 group, in the subject of Social Sciences. The class was made up of 27 boys and girls of different origins, most of them from outside Spain (Dominican Republic, Morocco, Poland, Georgia, Mali and China).

The classroom was spacious and bright. The teacher's desk was large and located at the side of the classroom. The students' tables were arranged individually. Although the styudents had a seating plan set by the teacher, we noticed that in this class students sat wherever they wished, a factor that seemed to affect the development of the class. The keener students sat closer to the teacher, while the more distracted students sat further away, at the back of the classroom. In our opinion, this seating arrangement reinforced the hierarchical difference between teacher and students.

II. Organisation of the observed activity

The class consisted of an explanation of the concepts of Marxism and anarchism by the teacher. The resources used were a computer, a projector and a digital book. The presentation lasted 1 hour. The method used by the teacher was instructive exposition, which took up most of the time and was followed by questions and answers to check and evaluate learning.

III. Development of the activity

The teacher explains to the whole class the main features of the two ideologies under study (Marxism and anarchism). She supports her explanations by reading excerpts from the digital book that she is projecting on the board. She reads the definitions out loud, highlights some information and/or adds further information. She shows photographs of Marx and Bakunin. At certain points during her presentation, the teacher asks probing questions such as: “what movement would you support? Why? Do you think these movements would make sense in current times? Do you know any communist country?”

Three quarters of class time are devoted to the teacher’s presentation of the topic. Very few students are paying attention, only two are taking notes. Those who are not listening are talking among themselves, insulting each other, throwing paper balls, listening to music on their mobiles or playing on their devices. In a resigned tone, the teacher asks the class to be quiet: “Come on, guys, be quiet ... Shhh”. The students ignore her.

As if nothing had happened, the teacher continues talking, using the digital book as support. A student plays with a ball at his feet, under the table. Now and then, the ball rolls off and he gets up to get it. The teacher gives him a sidelong look, without saying a word.

20 minutes before the end of the class, the teacher ends her presentation and she begins to ask the students questions, randomly. She addresses a student directly and asks him what movement he would support if he was a factory worker in the 19th century. Annoyed, the boy answers: “What are you going on about!" Outraged, the teachers hands down a penalty to the student: “That’s a penalty for you!” (*a penalty is a sanction for not observing class norms whish is then reported to school management and is later discussed in the school council*). The student reacts contemptuously, mumbling something under his breath.

Although there is still generalised unrest in class, the students have calmed down a little. The teacher continues with her questions and answers session. Some students provide better answers than others but, regardless, she encourages and praises everyone, giving brief feedback:” Well done!”, “That’s right!”. Now and then, she admonishes some students who are distracted: “Jessica, take your bag off the table”, “Roberto, put your mobile away or I’ll confiscate it”, “Xavier, be quiet!”. Some students seem offended, like Xavier, who retorts “Call me Xavi”. The teacher maintains an authoritarian tone: “I will not stop calling you Xavier unless you keep quiet”. Other students completely ignore her admonishments.

The commotion is almost generalised. Suddenly, a pupil throws a crumpled piece of paper into the bin, aiming from his seat. The paper falls to the floor and he gets up and throws it again from a distance. The teacher asks him to deposit the paper in the bin and to sit down at once. The pupil does so. When the boy sits back in his chair, she resumes the lesson, highlighting the correct answers.

The class ends abruptly when the bell rings. The students get up and leave the room, in a hectic manner.

************************ *** ***************************

Case B

Class observation and analysis of class dynamics in a Year 4 group (Author: FMP, teacher MGP) (December 2019)

I. Description of the context in which the learning activity takes place

The activity takes place at the secondary school XX [information omitted to maintain confidentiality], a setting of complex needs located in Santa Coloma de Gramenet (Barcelona), on Friday 1st December 2019 at 8:00 am.

The activity was part of a two-hour Social Sciences lesson, given to Class A of Year 4 of secondary education, and took place in the regular classroom. It should be noted that the school has four groups (A, B, C and D). Group A is the set for students with the most learning difficulties.

In terms of materials, support and resources, the classroom has an electronic whiteboard that is used during the activity, as well as several laptops, which are kept in a locked cabinet but can be used when the teachers require them for certain activities. In this classroom, students have their own notebooks, mostly with grid sheets, and pens so that they can take handwritten notes as necessary.

At the beginning of the class there are 14 students, six girls and eight boys. Five students are absent. All the students in this class group are children from families that were part of the new migration wave over the last two decades, and they come from very diverse family backgrounds: Morocco, Pakistan, Bangladesh, China, India and the Dominican Republic. One of the girls, who has just arrived from Romania, attends both the class group lessons and the Newcomers' Classroom. With the exception of one student who wears a veil, no distinctive clothing or accessories are observed. Nor are there any characteristic features of any urban tribe in their accessories.

There are two students seated per table, facing the blackboard. Most of the students are situated on one side, the one with the window and the heating. It is December, and winter is approaching.

II. Organisation of the observed activity

The activity observed is part of a didactic unit on the contemporary history of Catalonia and Spain. The aim of this didactic unit is to understand the major historical stages of the 20th century, both in our country and in Spain as a whole (Republic, Civil War, Francoism, Transition and Democracy). To do so, the Social Sciences teacher has planned a series of chronological sessions. The lesson under observation is the first one in this series and, in addition to providing an introduction to the whole didactic unit, it is the start of the study of the years of the Republic.

This session introduces the study topic by looking at the life of a working-class priest from Santa Coloma de Gramenet, from the parish of XXX [information omitted in order to maintain confidentiality], which is close to the school. Following the priest's life story, the chronology of the 20th century is presented as the context. In this way, the lesson is contextualised through the life of this historical figure, aiming to illustrate the years of the Second Republic: the dates of its beginning and end, its proclamation, the main characteristics of the period and the political protagonists who were at the head of the Spanish government and the Generalitat of Catalonia in those years.

The teacher used mainly an inquiry-based learning methodology in small groups. The lesson lasted 1 hour.

III. Development of the activity

The activity begins with the teacher's introductory explanation. This first thematic overview takes the form of a lecture. From 8:00 to 8:15 a.m., the teacher gives an overview of the life of Father José Sánchez, from his birth to his final days. Particular emphasis is placed on his social commitment and the crucial role he played during the second Franco regime and the transition years as a dynamic agent in a neighbourhood built up by the migration waves from the south of Spain in the 1960s.

The teacher gives a coherent and relatable biographical account, as if it were a story, which captivates the students, most of whom follow the explanations in silence. Some take notes in their notebooks. Even those students who in previous sessions showed little interest in the subject now seem to be interested in this topic.

Whilst telling the story, the teacher asks some questions about specific aspects of the neighbourhood in order to complement the explanation and interact with the students. The nameplate of a street, the place where the parish church is located, etc. The students respond actively, particularly those in the first row. Although the answers are not always correct, together and with the teacher's help, they manage to answer the questions that allow the biographical story to continue. The teacher also draws a chronological line on the electronic

blackboard and on it she places , in the right order, the historical periods of the 20th century: Republic, Civil War, Francoism, Transition and Democracy.

In order to begin to situate the different historical stages experienced by the priest and which have transformed the neighbourhood and the city, at 8.30 am the teacher asks the students to look for information on the Second Republic, with specific items (dates, relevant events and political protagonists). To complete this task, the teacher asks one of the students to distribute the laptops to his classmates, giving one to each pair of students. It is at this point that the classroom climate changes. Whilst until then the class was quiet and calm, it now turns into a hullabaloo, in the two minutes that pass while the computers are being handed out. Students start talking to each other, the tone of voice rises and some students get up from their chairs. After a few moments of movement, a dispute breaks out between two students, who confront each other for no apparent reason. They push and shout at each other. The teacher intervenes to restore order in the class. She does so by calling out to students individually and asking them to start working the activity, and asking if they have any questions.

For the next half hour, the student pairs search for the requested information, some more proactively than others. At 8.40 a.m., just as they were beginning their research, the fire alarm goes off. It was a false alarm, as the teacher on hall duty confirmed a little later. The students already knew. For three days now, someone has been setting off the alarm between classes, disrupting the normal functioning of the school. Even so, some students in the class group take advantage of the situation to raise their voices again and push tables around, making a lot of noise. The teacher, once again, has to restore order, and reminds students they could face detention and stresses the importance of not pressing, without a valid reason, any of the eight fire alarms buttons installed around the school.

Once calm has been restored, students continue working on the task. The teacher approaches students to help them identify relevant information. She gives individual attention especially to the newly arrived student. We see expressions of joy in those students who are finding the information. We also observe a couple who are not very interested in the activity and who are distracted by consulting content on the Internet that has nothing to do with the activity. Only when the teacher approaches them to ask if they have any queries do they start working.

The last 10 minutes of the class are spent sharing knowledge. The teacher asks the whole class about the different topics, and the most motivated students start answering the questions, although not always correctly, according to what they have found. As the minutes go by, the teacher also asks direct questions to those who have not yet participated. She congratulates students on correct answers, praises them and helps those with incorrect ones. The final answers are written down on the electronic whiteboard. All the information requested has been found. The work is done, and the bell rings. Again, there is a commotion. The students put away the laptops, making a lot of noise as the tables are being pushed, and they head out into the playground. “Kids, the Civic War next time!”, says the teacher loudly.

************************ *** ****************************
