# Supplementary material for: The Role of Emotions in Classroom Conflict Management. Case Studies Geared Towards Improving Teacher Training
Source: Front Psychol. 2022 Mar 16;13:818431. doi: 10.3389/fpsyg.2022.818431 (PMC8967289; doi:10.3389/fpsyg.2022.818431)
Supplement: Supplementary file 2 [file Data_Sheet_2.docx]

**Appendix 1. Class observation protocol for evaluating a learning activity**

| **Institution** |  | | |
| --- | --- | --- | --- |
| **School subject** |  | | |
| **School Year** |  | **Class** |  |
| **Teacher** |  | **Pre-service teacher** |  |

| Peer observation | Individual observation |
| --- | --- |

| **INTERACTIONS** | **Observed performance** | **Proposed improvement** |
| --- | --- | --- |
| **Start of the class** |  |  |
| The teacher encourages student to formulate and verbalise questions about the new content? |  |  |
| The teacher states or reminds students of the learning objectives, the reasoning behind them and how they will be met? |  |  |
| The teacher organises the work session, reminding students of class norms and justifying these or renegotiating them if required? |  |  |
| The teacher introduces tasks that allow students to start learning autonomously?  … |  |  |
| **During the class** |  |  |
| The teacher fosters a positive work environment, addressing negative aspects as they come up? |  |  |
| The teacher uses methodologies that allow for the inclusion of all students?  **…** |  |  |
| **End of the class** |  |  |
| The teacher spends some class time promoting reflective thinking and the verbalisation of the knowledge acquired? |  |  |
| The teacher asks questions such as: what have we done in class today? What have we learned? What did we set out to learn? What is still unclear? What worked best? |  |  |
| The teacher asks students to discuss what they have learning with their peers? |  |  |
| The teacher reflects with the students on their contribution to the satisfactory performance of their groups and any areas for improvement?  … |  |  |
| **PRINCIPLES FOR PROMOTING LEARNING** | **Observed performance** | **Proposed improvement** |
| 1. **Encourage contact between students and teachers** |  |  |
| Who starts the interactions?  Are students encouraged to take the lead in their learning? |  |  |
| Are students encouraged to commit actively to their learning and to understand their own role as learners? |  |  |
| Are students at any point given the chance to choose, propose or decide what to do next? |  |  |
| Is assessment always done by the teacher or are peers also involved?  … |  |  |
| 1. **Develop reciprocity and cooperation among students** |  |  |
| Are students’ tasks based on the social nature of learning? Is cooperative learning properly structured and actively encouraged? |  |  |
| What proportion of class time is devoted to group work, pair work and individual work? |  |  |
| Is the classroom space arranged and used in a manner that fosters interaction? How are the tables and chairs laid out? |  |  |
| How are class norms and class functioning decided on? |  |  |
| In what instances does the class become disrupted or in disarray? |  |  |
| What are the factors that intervene in the disruption? |  |  |
| What types of conflicts are more frequent? |  |  |
| How is disruption managed? How does the teacher respond? How do the rest of the students respond? |  |  |
| If the conflict is serious, what mechanisms does the teacher employ to deal with it?  … |  |  |
| 1. **Encourage active learning** |  |  |
| Does the teacher take into account students’ motivations and the key role that emotions play in achieving results? |  |  |
| Is teaching based on direct instruction or does it promote learning through enquiry? |  |  |
| Are problems solved or are procedures applied through exercises? Is dialogue promoted? |  |  |
| Does the class make use of textbooks, academic texts, journalistic texts, manipulative materials, audio-visual resources, web pages, etc.? |  |  |
| When and how are they used? What follow-up activities are done?  … |  |  |
| 1. **Give prompt feedback** |  |  |
| Have coherent evaluation strategies with an emphasis on formative feedback been applied? |  |  |
| Is evaluation understood as an open assessment based on guidelines and models? |  |  |
| Is individual self-assessment or co-assessment an option?  … |  |  |
| 1. **Emphasize time on task** |  |  |
| Have the class activities demanded from all the students a good effort and eagerness to excel, but without imposing an excessive workload? |  |  |
| Is the time devoted to each task sufficient to achieve the expected results? |  |  |
| Are resources used to facilitate the regulation of learning? |  |  |
| Is reflective thinking encouraged before, during and after the development of the tasks in order to adapt and agree on goals and procedures?  … |  |  |
| 1. **Communicate high expectations** |  |  |
| Before the start of a learning activity, is prior knowledge activated so that all students have the opportunity to experience the personal satisfaction of finding a solution? |  |  |
| Are there opportunities for students to check their learning progress? |  |  |
| Is explicit attention given to students who are falling behind or not completing tasks? Is help offered to enable them to find the answers for themselves? |  |  |
| Is positive feedback given?  … |  |  |
| 1. **Respect diverse talents and ways of learning** |  |  |
| Are the activity requirements formulated in a way so as to encourage all students to find their space for participation? |  |  |
| What strategies does the teacher use to encourage all students to learn, regardless of their differences? |  |  |
| What alternative resources are brought into play to foster the learning of all students?  … |  |  |
