# Supplementary material for: The Role of Emotions in Classroom Conflict Management. Case Studies Geared Towards Improving Teacher Training
Source: Front Psychol. 2022 Mar 16;13:818431. doi: 10.3389/fpsyg.2022.818431 (PMC8967289; doi:10.3389/fpsyg.2022.818431)
Supplement: Supplementary file 3 [file Data_Sheet_3.docx]

**Appendix 2.** Examples of codification of interactive segments of the cases analysed

S2b (Case A)

20 minutes before the end of the class, the teacher ends her presentation and she begins to ask the students questions, randomly. She addresses a student directly and asks him what movement he would support if he was a factory worker in the 19th century [AT] (rigid, centred on the content).

Annoyed, the boy answers: “What are you going on about!" [CI] (disinterest/open conflict).

Outraged [TEE] (disappointment, negative emotion), the teachers hands down a penalty to the student [TB] (Reactive orientation/Domination, use of position of power, involves the administration).

The student reacts contemptuously, mumbling something under his breath.

S5 (Case B)

Once quiet has been restored, the teacher approaches students to help them identify relevant information {…} [AT] (learner-centered paradigm). She notices that a student pair is getting distracted looking for content on the internet that have nothing to do with the task [CI] (distraction/potential conflict).

The teacher discreetly approaches them [TEE] (interest) and asks them if they have any questions [AT] (flexible, empathetic) [TB] (Active orientation/Collaboration/Open communication).

The students feel they’ve been caught out, they pretend nothing has happened and move on to work on the task.

Abbreviations

S= Interactive segment

TB = Teacher’s behaviour towards conflict

TEE = Emotional experience

AT = Attitud

CI = Critical Incident (conflict)

{…} = Modified information. Simplification of an expression while preserving the meaning
